# Contiguous Lumbar Bilateral Pars Defects at L3 and L4 (With Intact L5): Treated With Pedicle Screw Fixation and Interbody Fusion: A Case Report With Literature Review

**DOI:** 10.1002/ccr3.73556

**Published:** 2026-10-14

**Authors:** Muhammad Rizwan Ali, Muhammad Jawad Saleem, Ali Shahid, Muhammad Suliman Sajid, Muhammad Haris, Shayan Asmat, Noor ul Subah Afzal, Mohammad Al Diab Al Azzawi

**Affiliations:** ^1^ Department of Orthopedic Surgery Allama Iqbal Medical College Lahore Pakistan; ^2^ Department of Orthopedic Surgery Faisalabad Medical University Faisalabad Pakistan; ^3^ Faculty of Medicine The National Ribat University Khartoum Sudan

## Abstract

A 42‐year‐old woman had chronic L4 pars defect with Grade 2 spondylolisthesis and acute L3 pars fractures after trauma, causing back pain, sensory, and motor deficits. She underwent L3–L5 pedicle screw fixation with interbody fusion. Postoperatively, she showed rapid pain relief, neurological improvement, and stable spinal alignment on follow‐up.

## Key Clinical Message

Contiguous bilateral pars defects at adjacent lumbar levels caused by mixed acute and chronic mechanisms are extremely rare. Careful clinico‐radiological correlation is essential for diagnosis, especially when advanced imaging is limited. In unstable cases with neurological deficits, posterior pedicle screw fixation with interbody fusion provides effective decompression, stabilization, and favorable early functional recovery.

## 1 Introduction

Pars defects, burst fractures, and instability are commonly recognized pathologies. Fractures and defects of the lumbar spine are the common repercussions of trauma and degenerative changes. Pars interarticularis, which is a thin portion of the vertebral arch, is very vulnerable to stress‐related degeneration as well as traumatic injuries. The incidence of pars interarticularis defects is higher (about 4%–6%) in the athletic population and adults with repetitive extension loading [1, 2]. These defects can occur unilaterally or bilaterally, and their natural history varies from asymptomatic incidental findings to painful instability and progressive spondylolisthesis [3]. Isthmic pars defects (spondylolysis) are osseous defects of the pars interarticularis that may develop as stress fractures secondary to repetitive or chronic low‐grade mechanical loading and may progress to chronic inactive non‐union. [4]. Acute traumatic pars defects are caused by high‐energy mechanisms such as falls or road accidents and occur rarely [5]. However, the coexistence of a pre‐existing isthmic pars defect at one level and an acute traumatic pars defect at an adjacent vertebra is extremely rare. The case is also noteworthy because the pars defect was bilateral at L3 and L4 vertebrae arising from different mechanisms with intact L5.

We searched databases (PubMed, ScienceDirect, and Google Scholar) using relevant keywords Lumbar pars defect, Contiguous pars defects, L3 and L4 vertebrae, Pedicle screw fixation, Interbody fusion, case report, and Lumbar Pathology till 12th of June 2026. We found only some case reports of adjacent‐level lumbar pars defect, but to the best of our knowledge, neither described this type of lumbar pathology. Here, we discuss a rare case with bilateral pars defects at adjacent levels L3, L4 with intact L5, arising from different mechanisms which were managed surgically using pedicle screw fixation and interbody fusion. This article reviews the current literature on contiguous lumbar pars defects, using a rare clinical case as an illustrative example.

## 2 Case Presentation

A female of 42 years of age sustained a roadside accident. She was shifted to the emergency department in our hospital on a spine board by qualified paramedical staff. On arrival, she was conscious, oriented, and hemodynamically stable. She did not have any external injury or bony tenderness over limbs except for deep tenderness over the lumbar spine. On neurological examination, she had sensory deficits involving L3 and L4 dermatomes. While motor examination revealed weakness of bilateral hip flexion and knee extension (Grade 3/5). Reflexes were preserved, and there was no bladder or bowel incontinence.

### 2.1 Differential Diagnosis, Investigations and Treatment

She had a history of lower back pain for 2 years, secondary to an old L4 isthmic pars defect with no other significant comorbidities. According to the Wiltse‐Newman classification, the L3 pars defect was characterized as Type II C and the L4 pars defect was marked as Type II A. Radiographical examination including AP and lateral views (with extension and flexion) showed that she was found to have a chronic pars defect at L4 with Grade 2 anterolisthesis (isthmic in nature), acute pars defect contiguous at L3, consistent with trauma, while L5 was intact without any evidence of fracture as shown in Figure 1.

**FIGURE 1:**
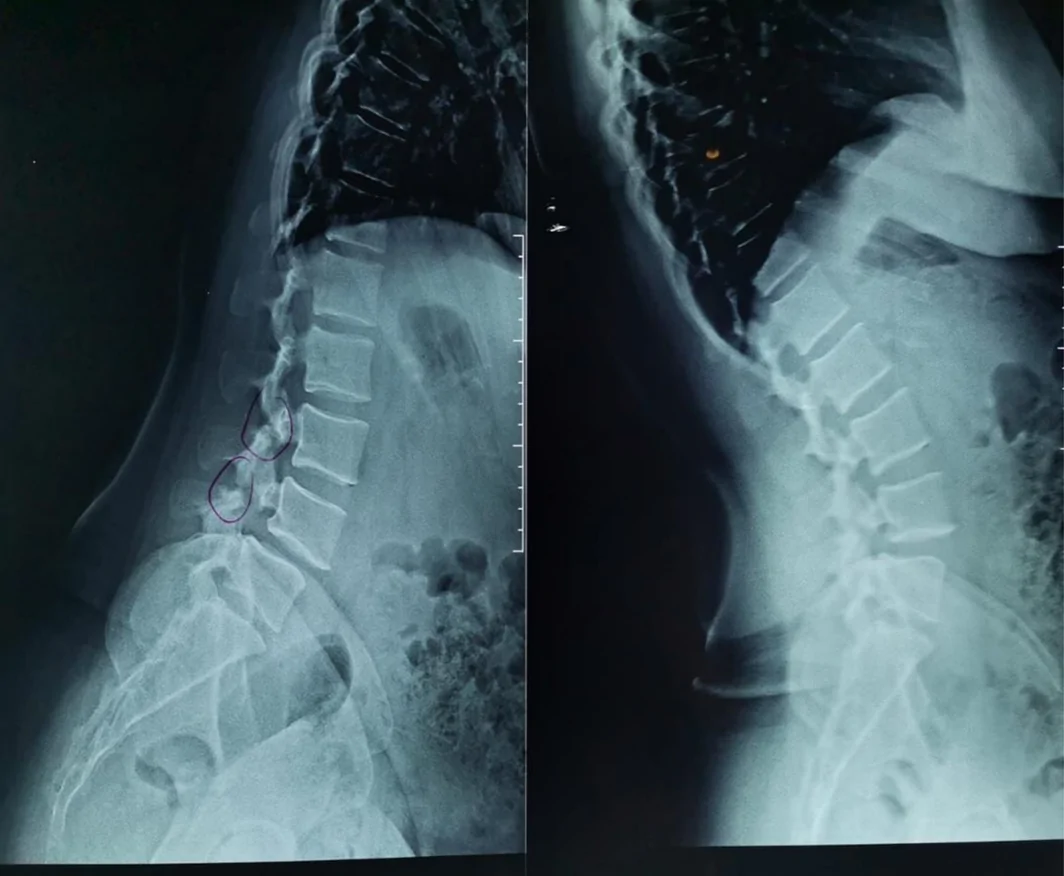
Lateral view of x‐rays of Lumbosacral vertebrae with flexion and extension respectively.

As the case was presented in the emergency department and due to institutional resource limitations at the time of admission of the patient, we could not obtain a preoperative MRI and CT scan. We distinguished acute and chronic pars defects by clinico radiological correlations which includes history of patients of acute high energy trauma, a two‐year history of low back pain, dynamic radiographs, and intraoperative findings. The L3 pars defect showed features that were consistent with an acute injury. While L4 pars defect showed fibrotic margins which suggests it to be a chronic isthmic process (Figures 2 and 3). CT and MRI would have provided superior characterization of pars morphology, lesions chronicity, neural element compression, and associated soft tissue injury.

**FIGURE 2:**
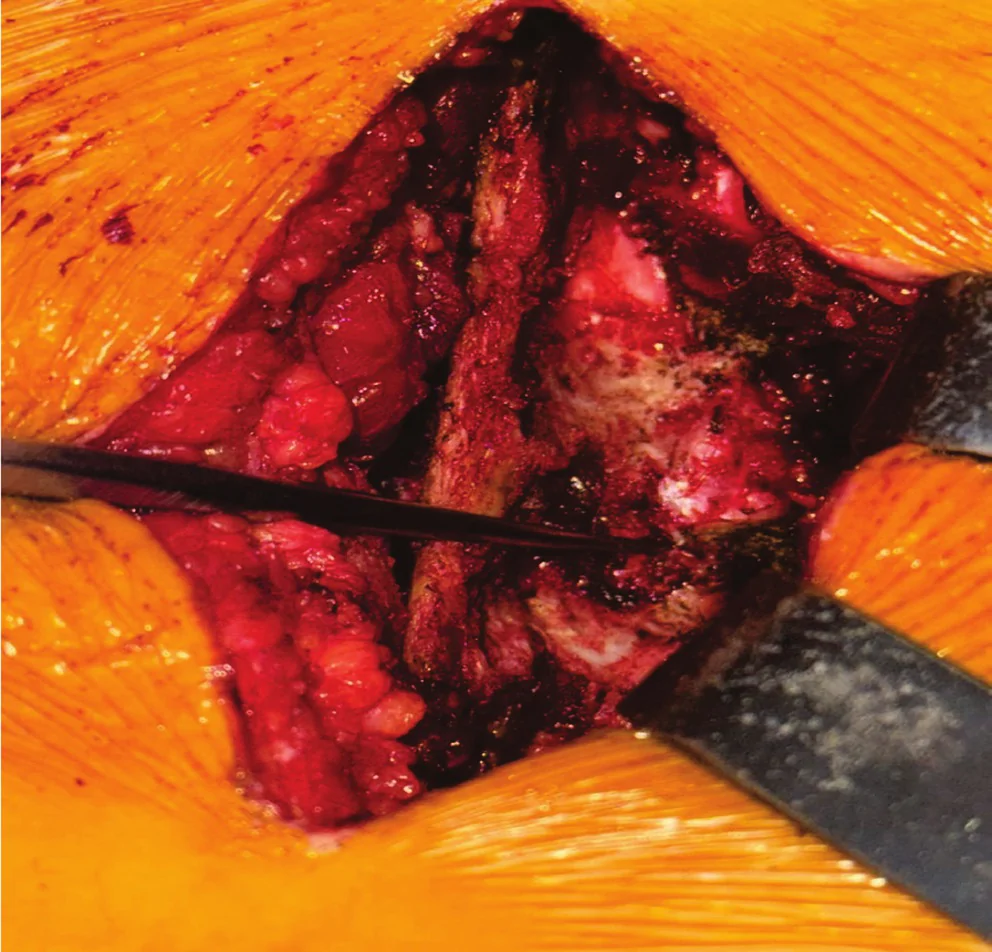
Intraoperative photographs.

**FIGURE 3:**
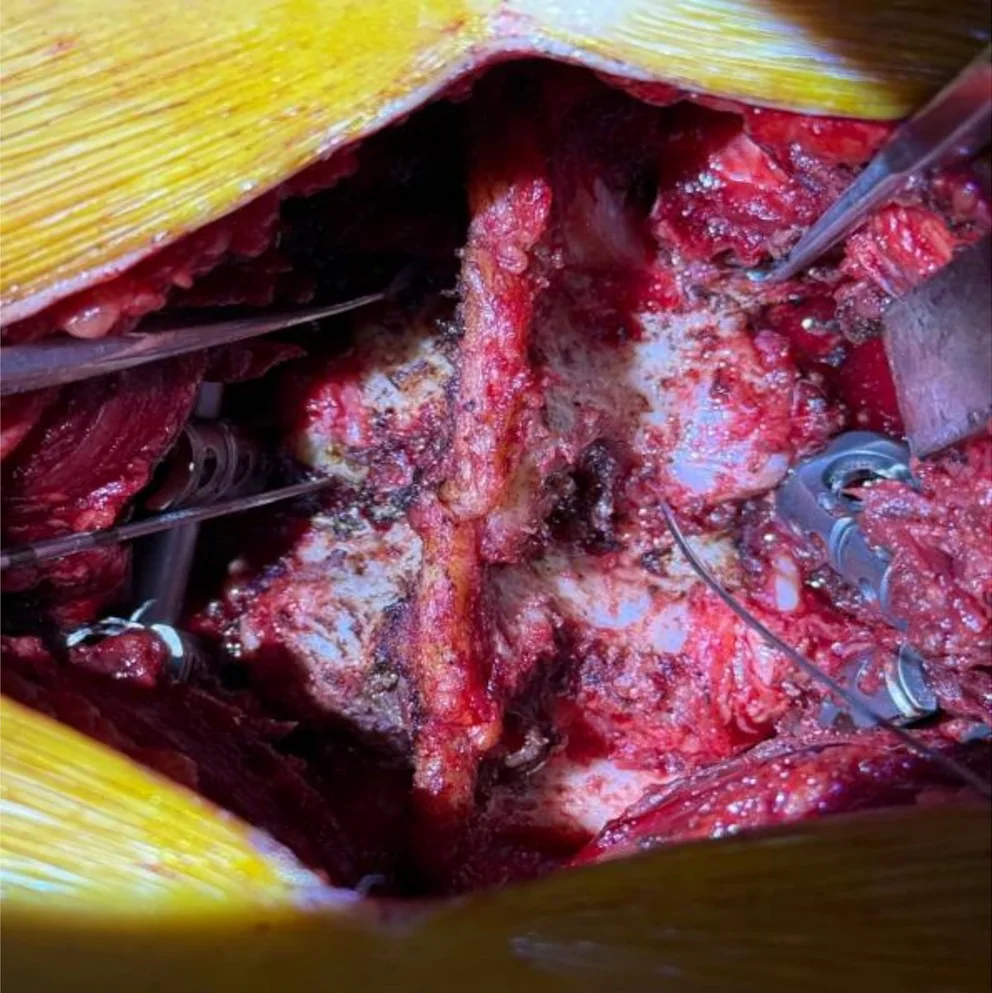
Intraoperative photograph representing 4 pars defects simultaneously which are being fixed by pedicle screw fixation and interbody fusion.

The patient was planned for posterior stabilization and fusion under general anesthesia with intraoperative neuromonitoring. A midline posterior approach from L3 to L5 with subperiosteal dissection of paraspinal muscles exposing the posterior elements. Under fluoroscopic guidance, pedicle screws (6.5 × 45 mm) were inserted bilaterally at L3, L4, and L5. To restore and maintain sagittal alignment, rods were contoured with optimal lordosis. Adequate decompression was achieved by laminectomy, flavectomy, medial parsectomy, and foraminotomy of L3–L5. At L3–L4 and L4–L5 levels, right‐sided facetectomy, cruciate annulotomy, disc excision, and preparation of the end plates were performed, followed by insertion of interbody PEEK cages packed with bone graft and bone substitute. Imaging was used to confirm adequate placement of instrumentation and cages, hemostasis was secured, and the wound was closed in layers over a drain followed by aseptic dressing (Figures 4 and 5). We did not consider isolated pars defect repair and conservative management techniques because of the presence of neurological deficits, Grade II anterolisthesis at L4, and presence of instability at two contiguous levels. So, our surgical goal was to achieve adequate decompression, restore sagittal alignment, and provide rigid stabilization across unstable segments. We considered direct pars repair but did not proceed with it because it was considered unsuitable because of the chronic L4 defect with Grade II spondylolisthesis, presence of instability at two levels, and neurological compromise which requires decompression. A shorter construct would neither address both defective levels adequately nor provide sufficient biomechanical stability following decompression. Therefore, we selected L3–L5 fixation with two interbody fusion to achieve durable stabilization and restoration of spinal alignment.

**FIGURE 4:**
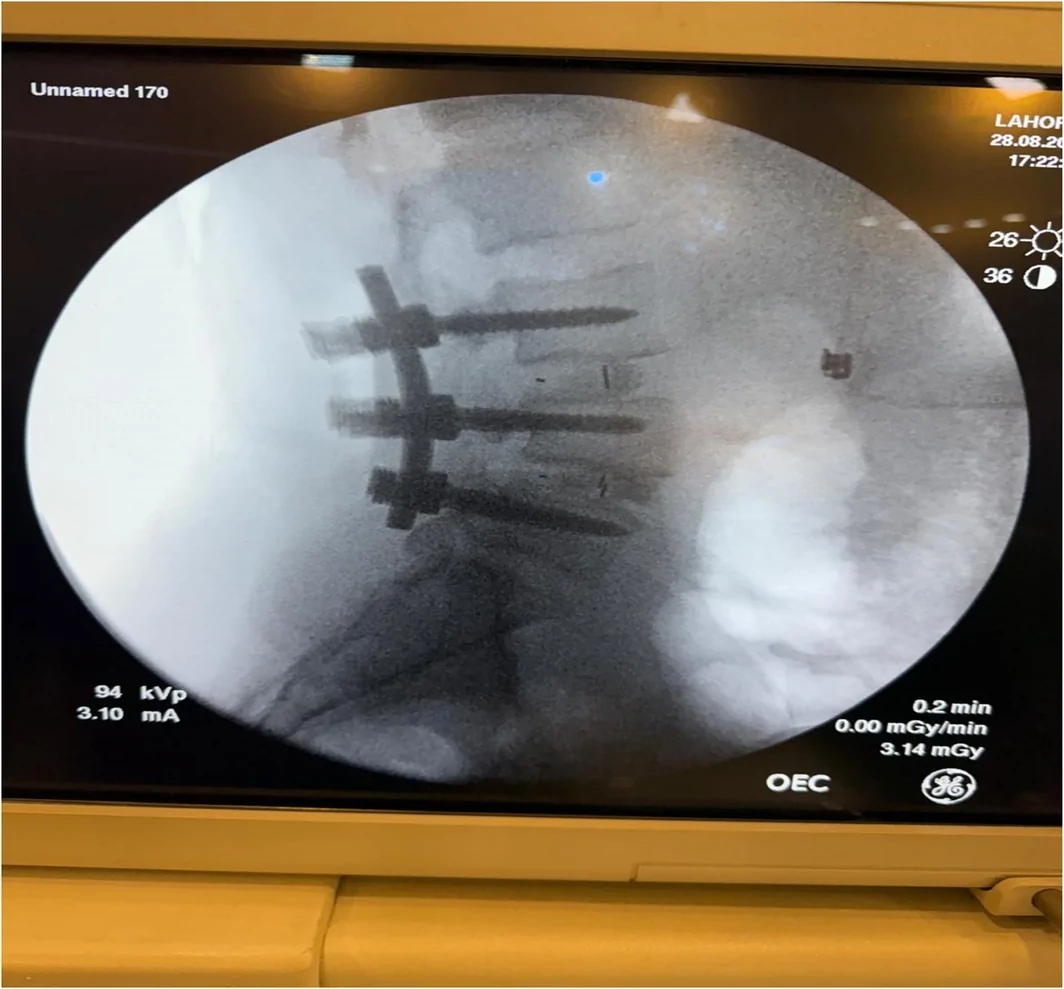
Intraoperative lateral fluoroscopic image demonstrating pedicle screw fixation across L3 and L4 vertebrae for stabilization of contiguous bilateral pars defects. Six screws are visible, two on each side, connected by rods, confirming correct alignment and hardware placement.

**FIGURE 5:**
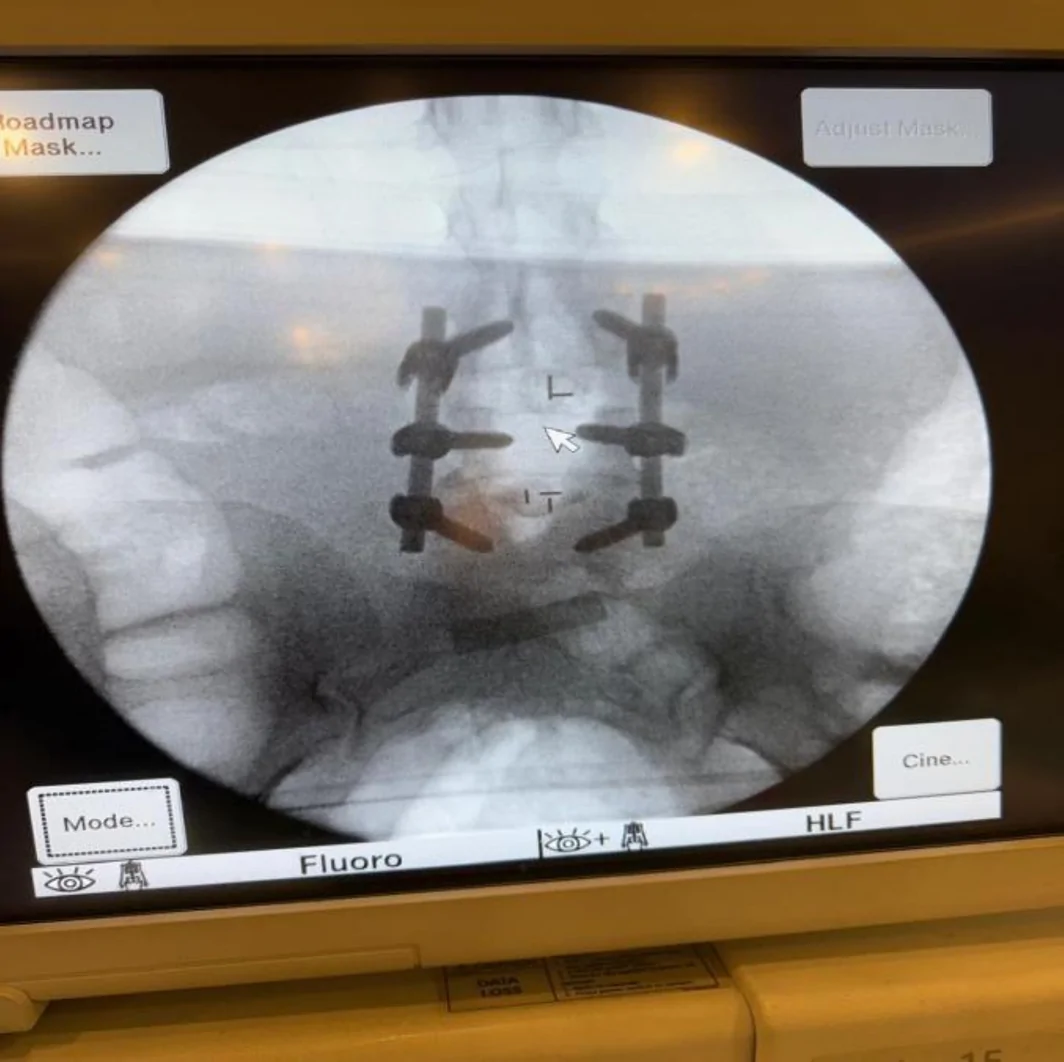
Intraoperative fluoroscopic image showing pedicle screw fixation at L3 and L4 levels for stabilization of contiguous bilateral pars defects with intact L5 vertebra.

The patient was shifted to the recovery room and then to ward with stable vitals. The drain output was 100 mL with intact dressing as well as good pain control. The patient was out of bed and mobilized first post OP Day and was discharged the next day after removal of the drain.

Postoperatively, the patient was followed for 3 weeks (29 August 2025) and again at approximately 10 weeks (17 October 2025). At the first follow‐up, the wound healing was satisfactory, and stitches had been removed (Figure 6). The patient reported much improvement in her back pain. The visual analogue scale score decreased from 8/10 to 2/10. On neurological examination, there was gradual recovery of sensations in the affected dermatomes. Moreover, motor strength improved from 3/5 to 5/5. Plain radiographs at 3 weeks showed good spinal alignment and stable hardware positioning. At the second follow‐up, the patient remained neurologically intact, ambulatory, and independent in daily activities without any complications. Radiographs showed maintained alignment, stable instrumentation, and ongoing progressive fusion at L3–L4 and L4–L5 interbody levels (Figure 7). No evidence of hardware loosening, implant failure, or loss of correction was observed.

**FIGURE 6:**
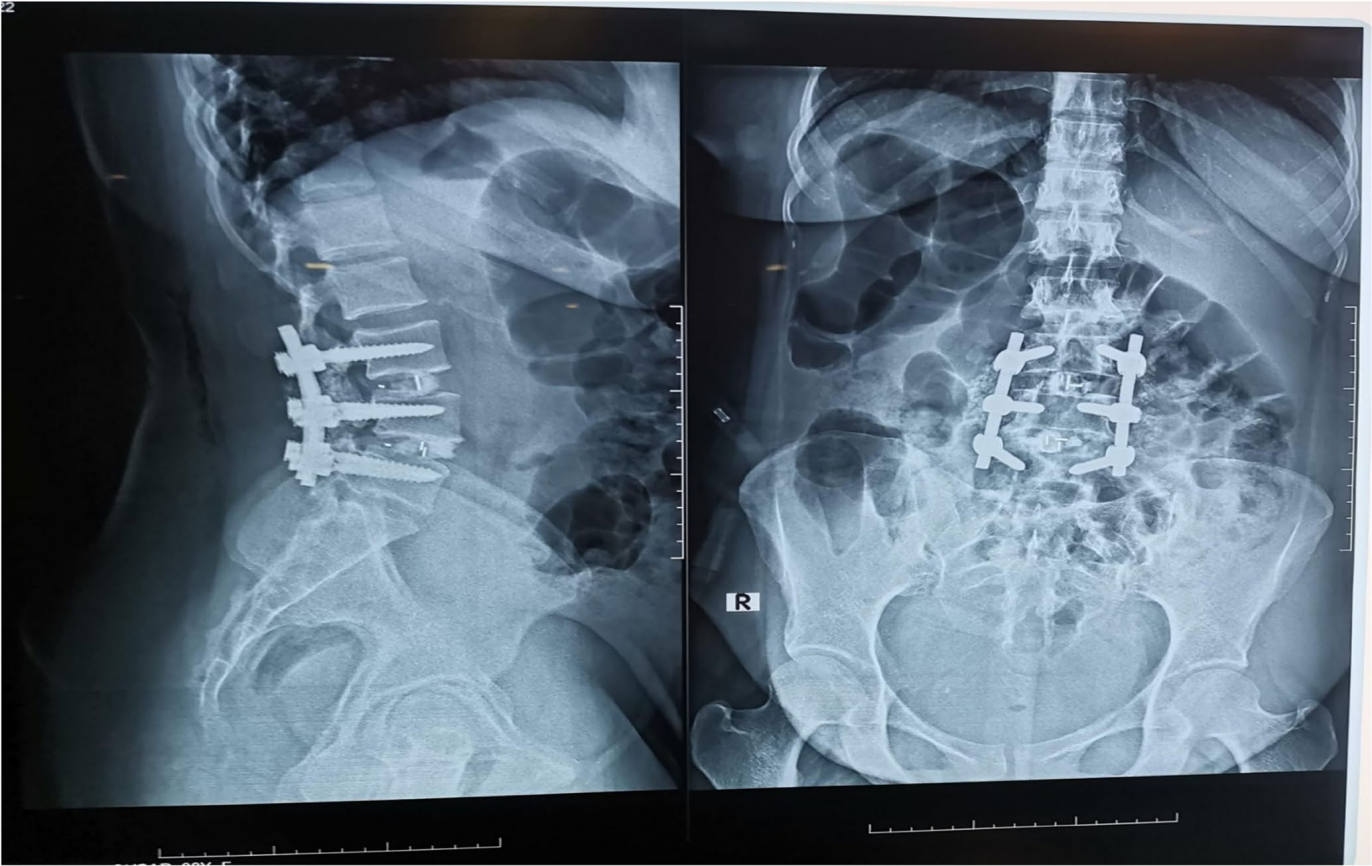
Post OP X‐rays in Lateral and AP view at 3 weeks.

**FIGURE 7:**
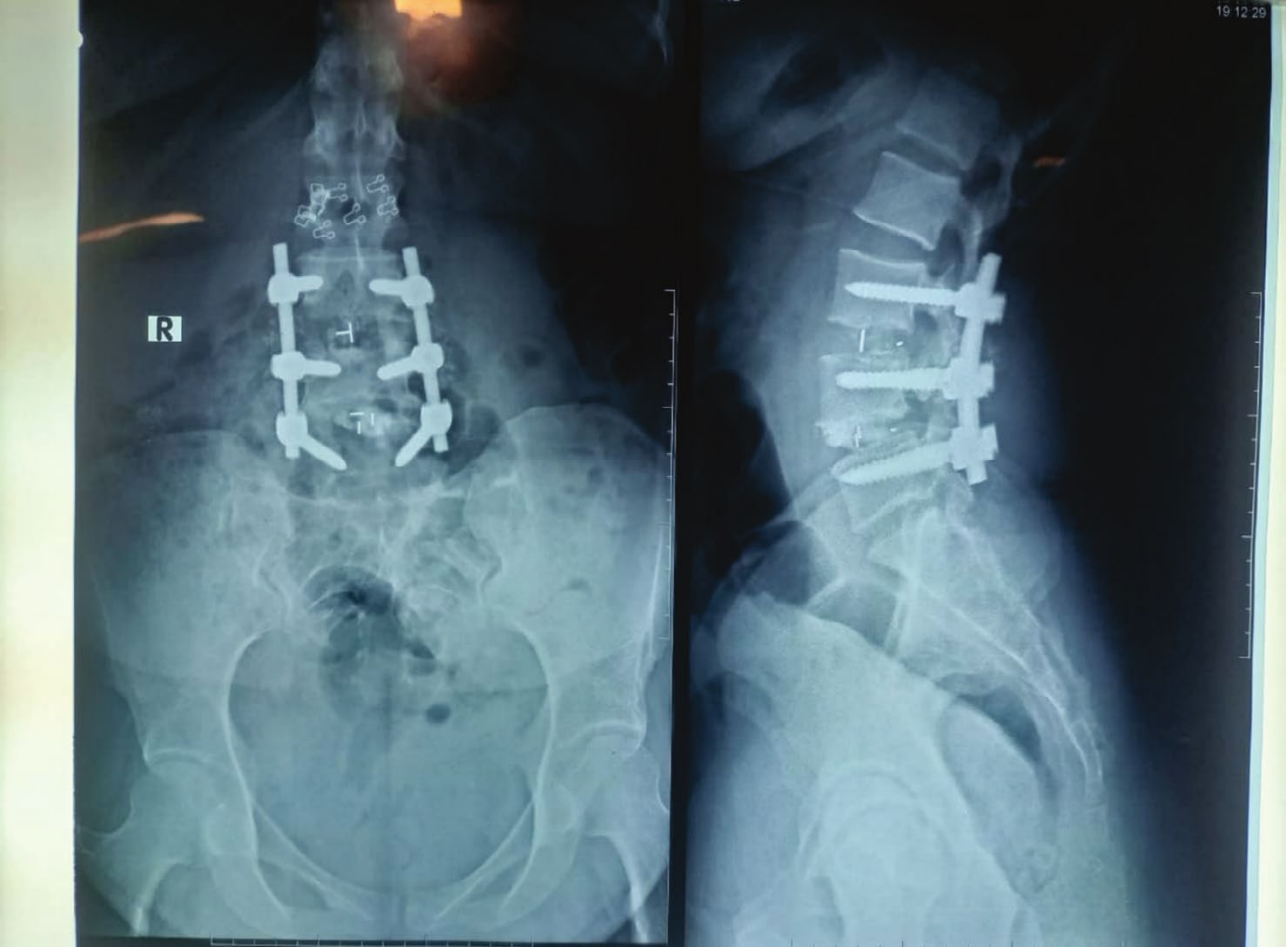
X‐rays at 10 weeks showing stable alignment and progressive fusion.

## 3 Discussion & Review of Literature

Fractures of pars interarticularis are believed to be clinically significant because they compromise the stability of the vertebral arch structurally. Complete fractures of the pars interarticularis may lead to anterolisthesis of the affected vertebra relative to the vertebra immediately inferior to it (spondylolisthesis), at which point neurological symptoms may occur [6]. Isolated isthmic and traumatic pars defects are well described in the literature, but the occurrence of contiguous bilateral pars defect at adjacent levels which arise from different mechanisms remains exceptionally rare.

Isthmic pars defects are generally considered to be the result of repetitive mechanical stress and fatigue loading of the pars interarticularis. They are particularly observed in the lower lumbar spine, being most frequently at L5 followed by L4. It often evolves gradually and sometimes it remains asymptomatic until instability or spondylolisthesis develops. In contrast, acute traumatic pars fractures are uncommon and are typically associated with high‐energy mechanisms such as motor vehicle accidents or falls. These injuries may be easily overlooked in the acute trauma setting especially when conventional radiographs are the primary imaging modality. The coexistence of these two distinct pathological processes at adjacent vertebral levels, as seen in our patients, represents an unusual injury pattern that is rarely reported in existing literature.

This case highlights the importance of maintaining a high degree of suspicion for atypical lumbar injury patterns in trauma patients with a history of chronic back pain. The absence of preoperative CT and MRI imaging represents a limitation; however, careful clinicoradiological correlation, dynamic radiographs, and intraoperative findings allowed us to differentiate between acute and chronic defects. This highlights the continued significance of thorough clinical assessment and surgical judgment in environments where advanced imaging may be unavailable. From a surgical perspective, management of multilevel pars pathology remains controversial because direct pars repair preserves motion segments in selected patients and preserves disc integrity. However, in our case, the coexistence of Grade II spondylolisthesis, neurological deficits, and instability at contiguous levels made us perform instrumental fusion. Compared with direct pars repair, fusion provided immediate stability and facilitated neural decompression though it sacrifices motion segments.

A review of the existing literature demonstrated that pars defects mostly involve the L5 vertebra, followed by L4, with involvement of L3 being very rare. Multilevel or contiguous pars defects are rare. They are predominantly reported as stress‐related or degenerative defects particularly in young athletes. Published reports that describe adjacent‐level pars defects are largely limited to isolated case reports and small case series. These typically involve similar underlying mechanisms at each affected level. Importantly, we were unable to identify prior reports that describe contiguous bilateral pars defects at L3 and L4 which arise from different mechanisms, namely an acute traumatic defect adjacent to a chronic isthmic defect with preservation of the L5 vertebra. To further evaluate novelty claim, we performed an updated literature review of PubMed, Google Scholar, and ScienceDirect till 12th of June 2026 literature by using combinations of the terms “multilevel pars defect,” “adjacent fracture,” lumbar spondylosis', and “contiguous pars defect.” Although several reports reported multilevel defects, none reported any case of L3 and L4 contiguous bilateral pars defect arising from different mechanisms (Table 1 showing spectrum of Pars Defect). This suggests that the injury pattern observed in our patient represents an exceptionally rare biomechanical scenario within lumbar spine pathology (Summary of Literature on pars defect is given in Tables 2 and 3).

**TABLE 1:** Showing spectrum of lumbar pars defects reported in the literature compared with the present case.

Characteristic	Typical single‐level spondylolysis	Multilevel spondylolysis	Contiguous adjacent‐level pars defects	Acute traumatic pars fractures	Present case
Most common level	L5	L5 ± L4	Usually L4–L5 or multiple adjacent levels	Variable	L3 and L4
Frequency	Common	Rare	Very rare	Rare	Exceptionally rare
Etiology	Repetitive stress/microtrauma	Stress‐related	Usually same mechanism at all levels	High‐energy trauma	Mixed etiology (acute traumatic L3 + chronic Isthmic L4)
Laterality	Often bilateral	Usually bilateral	Usually bilateral	Unilateral or bilateral	Bilateral at both levels
Age group	Adolescents/young athletes	Young athletes	Young adults/athletes	Any age after trauma	42‐year‐old female
Associated spondylolisthesis	Common	Common	Variable	Uncommon	Grade II L4 anterolisthesis
Neurological deficit	Uncommon	Uncommon	Variable	Possible	Present
L5 involvement	Usually involved	Frequently involved	Frequently involved	Variable	L5 preserved
Diagnostic modality	CT/MRI	CT/MRI	CT/MRI	CT/MRI	Radiographs + intraoperative findings
Treatment	Conservative/direct repair	Repair or fusion	Repair or fusion	Stabilization if unstable	L3–L5 fixation with two‐level TLIF
Key distinguishing feature	Single chronic defect	Multiple stress‐related defects	Adjacent defects with same pathology	Pure traumatic injury	Contiguous bilateral defects arising from different mechanisms with intact L5

**TABLE 2:** Showing comparative literature review table.

Author (year)	Levels involved	Etiology	Laterality	Associated spondylolisthesis	Treatment	Distinctive features	Difference from present case
Park et al. (2009) [19], PMID 20404945	L1–L4 (asymmetric: left L2–3–4, right L1–2–3)	Non‐traumatic, developmental/genetic + mechanical	Asymmetric bilateral (different levels each side)	None (lumbar kyphosis/instability instead)	Posterolateral fusion + pedicle screws L2–L4	4‐level asymmetric spondylolysis in a young athlete	Single uniform (non‐traumatic) etiology across all levels; no neuro deficit; L5 not the spared level here
Liu et al. (2015) [20], PMID 25525958	Mostly L3–L5, 2–3 levels (13 Patients)	Sports/trauma/heavy labor—but uniform per patient	Bilateral	Yes, 1–2 levels in all patients (mostly L4)	Pedicle screw fixation + interbody fusion, or direct repair	Largest reported series of multilevel spondylolysis with listhesis	Each patient has one mechanism throughout; none combine an acute fracture at one level with a chronic defect at the next, with L5 spared
Hersh et al. (2011) [21], PMID 22196393	L3, L4, L5 (bilateral pars defects)	Degenerative/isthmic (chronic)	Bilateral	Not specified	L4–S1 interbody fusion + direct repair of L3	First to combine fusion and direct repair at adjacent levels	Same chronic mechanism at all three levels; present case has acute (L3) + chronic (L4) mechanisms with L5 untouched
Darnis et al. (2014) [22], PMID 24657151	L3, L4, L5 (bilateral isthmic lysis)	Isthmic (chronic)	Bilateral	None	Combined anterior–posterior approach; bilateral L3 & L4 direct repair via L5–S1	3‐level involvement without any spondylolisthesis	Single chronic mechanism throughout, no listhesis, no neuro deficit; present case has Grade II listhesis and neurological involvement
Arai et al. (2013) [23], PMID 23607020	L3–L5 (3‐level spondylolysis)	Chronic (> 20‐year history)	Bilateral	None reported	Direct repair (pedicle screw–hook–rod), all 3 levels	Hardware impingement between L3 and L4 implants; L3 hardware removed after union	Motion‐preserving direct repair for chronic single‐mechanism disease, no acute component, no neuro deficit
Chen et al. (2024) [24], PMID 39574019	Bilateral pediculolysis L3/L4 + right spondylolysis L5	Degenerative/spontaneous, nontraumatic (osteopenic, 55M)	Bilateral (L3/L4) + unilateral (L5)	Instability/scoliosis at L3–L4; L5–S1 stable	L2–L5 PLIF + TLIF (long‐segment fusion)	Pedicle (not pars) involvement; proximal junctional failure at 2 years	Different bony structure affected (pedicle vs pars), single degenerative mechanism, longer/more extensive construct
Berger et al. (2020) [25], PMID 32953331	L4 (bilateral)	Acute traumatic (single level)	Bilateral	None	Conservative—bilateral steroid injections, no surgery	Acute traumatic pars fracture managed nonoperatively in an athlete	Single level, purely traumatic, no surgery; present case is two adjacent levels with mixed acute + chronic pathology and surgical fusion
Wu et al. (2016) [26], PMID 29252719	L3, L4, L5 (bilateral pars defects)	Old/childhood‐onset, ~30‐year history	Bilateral	Grade I at L4/L5 and L5/S1	Conservative (NSAIDs + 8‐wk physiotherapy)	29‐year radiological follow‐up of stable nonoperative disease	Chronic, low‐grade, asymptomatic‐for‐decades disease without neuro deficit; present case is acute, Grade II, with neuro deficit requiring urgent fusion
McKeeman et al. (2024) [27], PMID 39388987	L3–4 and L4–5 (double‐level isthmic spondylolisthesis)	Isthmic, same mechanism at both levels	Bilateral	Yes, Type IIA at both levels	L3–4/L4–5 Gill laminectomy + TLIF + L3–L5 posterior instrumented fusion	Closest surgical construct to present case; neurologic symptoms present	Both levels share identical (Type IIA) etiology and both have listhesis; present case has a different Wiltse type at one level (IIC at L3 but IIA at L4), with listhesis confined to L4 and L5 spared
Present case	L3 and L4 (bilateral); L5 intact	Mixed: acute traumatic fracture (L3, Type IIC) + chronic Isthmic defect (L4, Type IIA)	Bilateral at both L3 and L4	Grade II anterolisthesis, L4 only	L3–L5 pedicle screw fixation + 2‐level (L3–4, L4–5) TLIF	Contiguous bilateral pars defects at adjacent levels arising from two different mechanisms, with L5 spared	—

**TABLE 3:** Summary of literature on lumbar pars defects.

References	Year	Particularity of observation	Remarks	PubMed ID
Sakai et al. [1]	2010	Incidence and etiology of lumbar spondylolysis, most commonly at L5 followed by L4	Upper lumbar (L3) involvement is rare	20559793
Wiltse et al. [6]	1976	Classification of spondylolysis and spondylolisthesis	Foundational and widely used classification	1277669
Standaert & Herring [3]	2000	Critical review of spondylolysis mechanisms and presentation	Highlights stress‐related etiology	11131228
Leone et al. [4]	2011	Radiologic review of lumbar pars defects	Multilevel pars defects are uncommon	20440613
Gregory et al. [2]	2004	Sports‐related spondylolysis comparison	Supports repetitive stress mechanism	15562169
Sairyo et al. [17]	2006	MRI findings for early diagnosis of pars defects	Focuses on early stress injury	16418642
Mick et al. [28]	1993	Burst fractures of L5 vertebra	Isolated L5 injury is rare	8235876
Gurwitz et al. [12]	1993	Biomechanical analysis of lumbar fixation methods	Supports posterior instrumentation	8367785
Kaneda et al. [29]	1984	Anterior decompression and stabilization	Alternative surgical strategy	9010188
Luk et al. [30]	1986	Biomechanics of iliolumbar ligament	Explains stability of L5–S1	3958002
Chow et al. [8]	1989	Torsional stability of lumbosacral junction	Protective role of L5–S1	2749377
Pool‐Goudzwaard et al. [9]	1999	Anatomy of iliolumbar ligament	Resistance to traumatic injury	11693306
Sugawara et al. [10]	2016	Facet orientation and lumbar stability	Biomechanical explanation for intact L5	27790316

The possible cause of L5 to remain intact might be due to the result of a pre‐existing chronic isthmic pars defect at L4 which could have altered local spinal biomechanics and potentially acted as a “weak link,” absorbing and dissipating a part of the traumatic force. Moreover, the lumbosacral junction from L5–S1 is more stable due to the orientation of its facets and the support of sacrum and iliolumbar ligaments [7, 8, 9, 10]. It might be possible that the pelvis may have absorbed and redistributed forces at L5–S1. This may have reduced the transmission of axial load to L5, protecting it from injury. But its preservation remains speculative. Thus, due to these reasons, motion and stress were transferred cranially rather than caudally. However, these proposed mechanisms are hypothetical and should be interpreted cautiously because no biomechanical analysis was performed which can support these.

The treatment goals for patients with lumbar spinal injuries are to maintain or restore spinal alignment and stability, decompression to promote neurologic function, mobilize the patient, and start rehabilitation as soon as possible [5, 11]. Surgical treatment options for management include a posterior alone, anterior alone, or combined anterior and posterior approaches [12, 13, 14, 15], and lateral approaches. Long segment posterior stabilization and fusion surgeries should be avoided to conserve motion segments in the lumbar spine [16]. Although there are multiple minimally invasive techniques such as MIS‐TLIF, lateral lumbar interbody fusion, and motion preserving strategies, we performed TLIF because of limited institutional resources. Motion preserving strategies may reduce the risk of adjacent segment degeneration, but we favor performing fusion in this case because of established instability, neurological compromise, and spondylolisthesis with associated degenerative changes. Our primary goal was to achieve durable stability and neurological recovery, which outweighed the theoretical risk of adjacent segment disease.

Biomechanically, once a vertebra has developed a pars defect, the load transfer across the lumbar spine is altered, increasing motion and stress at adjacent segments. Less axial force is absorbed by the compromised segment. This makes the spine vulnerable to injury, particularly at the neighboring vertebrae [17]. But it is unusual for a subsequent trauma to produce a new pars defect at L3 while the caudal one (L5) remains intact, especially in the presence of a pre‐existing degenerative pars defect at L4. This combination makes it noteworthy [18].

The intact L5 vertebrae in our patient deserves particular attention. The lumbosacral junction is known to possess greater intrinsic stability because there is a coronal orientation of the L5–S1 facet joints, the support of the iliolumbar ligaments, and the structural role of the sacrum and pelvis. Most published case reports narrate contiguous fractures or stress fractures at adjacent levels; however, in our case, there is an atypical mechanism in which both the pars defects are arising from different mechanisms and are bilateral at L3 and L4 with intact L5.

## 4 Limitations

This case has certain limitations. Preoperative CT and MRI imaging were not available which reduces diagnostic certainty regarding the precise morphology, chronicity, and neural involvement of the pars defect. Although the follow‐up was extended to 10 weeks after surgery, this duration remains insufficient to have a definitive assessment of long‐term fusion success, implant durability, adjacent segment degeneration, and sustained functional outcomes. Long‐term radiographic and clinical surveillance is required. The biomechanical preservation of the L5 pars remains speculative because biomechanical studies were not performed. Despite these limitations, the rarity of the injury pattern and the favorable early clinical and radiological outcomes provide valuable insights into the diagnosis and management of complex lumbar pars defects in trauma settings, particularly in resource‐limited environments.

## 5 Conclusions

This report contributes to the limited body of literature describing contiguous lumbar pars defects and introduces a rare biomechanical scenario in which Chronic Isthmic and traumatic mechanisms coexist at adjacent levels (L3 and at L4) with intact L5. Awareness of such patterns is essential for timely diagnosis and appropriate surgical planning. The successful neurological recovery and short‐term radiological stability observed in our patient suggest that posterior pedicle screw fixation with interbody fusion is an effective treatment option for managing complex, unstable lumbar pars defects arising from mixed etiologies.

## Author Contributions

**Muhammad Rizwan Ali:** conceptualization, investigation, writing – original draft, visualization. **Muhammad Jawad Saleem:** conceptualization, writing – review and editing, data curation, methodology, project administration, supervision. **Ali Shahid:** validation, visualization. **Muhammad Suliman Sajid:** methodology, writing – review and editing, investigation, software, data curation. **Muhammad Haris:** visualization, methodology. **Shayan Asmat:** investigation. **Noor ul Subah Afzal:** investigation. **Mohammad Al Diab Al Azzawi:** validation.

## Funding

The authors have nothing to report.

## Consent

Written informed consent from the patient was obtained according to journal guidelines.

## Conflicts of Interest

The authors declare no conflicts of interest.

## Data Availability

The data that support the findings of this study are available from the corresponding author upon reasonable request.
